# COGNITIVE AGING AND SUCCESSFUL AGING: THE ROLE OF MEMORY

**DOI:** 10.1093/geroni/igac059.2084

**Published:** 2022-12-20

**Authors:** Neyda Ma Mendoza Ruvalcaba, Elva Dolores Arias-Merino, Melina Rodríguez Díaz, Maria Elena Flores-Villavicencio

**Affiliations:** UNIVERSITY OF GUADALAJARA, Guadalajara, Jalisco, Mexico; Universidad de Guadalajara, CUCS, Maestría en Gerontología, Guadalajara, Jalisco, Mexico; Universidad de Guadalajara, CUCS, Maestría en Gerontología, Guadalajara, Jalisco, Mexico; Universidad de Guadalajara, CUCS, Maestría en Gerontología, Guadalajara, Jalisco, Mexico

## Abstract

The cognitive functioning as a general measure, is a criterion commonly used to define and operationalize successful aging(SA). This study aims to explore the specific contributions of the memory and meta-memory to understanding successful aging (SA).(Project-Conacyt-256589)Population based, random sample included n=656 community-dwelling older adults 60-years and older (mean age=72.8, SD=7.6 years, 58% women). Memory was measured with the Digit span test backward (WAIS-IV). and meta-memory through self-report. Objective SA was operationalized as no important disease, no disability, physical functioning, cognitive functioning, and being actively engaged. Subjective SA was an appreciation if they considered themselves as successful agers. Sociodemographic and health data were also asked. Pearson′s correlation test and MANOVAs were performed.In total 11.2% met the criteria for SA, although 76% considered themselves as successful agers. 54.8 has a bad perception of their own memory, while 41.9% have objective decline. Results of the multiple regression analysis emerged on a significant model using the entered method:F=1.13,p<.01, explaining 14.9% of the variance of SA. Specifically, memory and meta-memory explained 19.5% of the variance of objective and 14.4% od subjective SA, 18.4% of the variance in the criterion of life engagement. Knowledge generated by this study reveals the specific role of the memory and metamemory on the SA, both objective and subjective. Besides, sets a scenario to promote successful and healthy aging, through alternatives centered in the improvement of memory and the judgment that is made about the own memory in older adults.

